# Poor outcomes for trial‐ineligible patients receiving polatuzumab for relapsed/refractory diffuse large B‐cell lymphoma in routine care: An Australian Lymphoma and Related Diseases Registry project

**DOI:** 10.1002/jha2.870

**Published:** 2024-02-23

**Authors:** Briony Shaw, Eliza Chung, Cameron Wellard, Edward Yoo, Rory Bennett, Callum Birks, Anna Johnston, Chan Y Cheah, Nada Hamad, Jock Simpson, Allison Barraclough, Matthew Ku, Nicholas Viiala, Sumita Ratnasingam, Tasman Armytage, Tara Cochrane, Geoffrey Chong, Denise Lee, Kate Manos, Colm Keane, Stephanie Wallwork, Stephen Opat, Eliza A. Hawkes

**Affiliations:** ^1^ Department of Haematology Monash Health Clayton Australia; ^2^ School of Public Health and Preventive Medicine, Monash University Melbourne Australia; ^3^ Department of Haematology Sir Charles Gairdner Hospital Perth Australia; ^4^ Department of Haematology Peter MacCallum Cancer Centre Melbourne Australia; ^5^ Concord Hospital Sydney Australia; ^6^ Department of Clinical Haematology Royal Hobart Hospital Hobart Australia; ^7^ Medical School, University of Western Australia Nedlands Australia; ^8^ Department of Haematology St Vincent's Hospital Sydney Australia; ^9^ School of Clinical Medicine, Faculty of Medicine and Health UNSW Sydney Australia; ^10^ School of Medicine University of Notre Dame Australia Sydney Australia; ^11^ Department of Haematology Port Macquarie Base Hospital Port Macquarie Australia; ^12^ Department of Haematology Fiona Stanley Hospital Perth Australia; ^13^ Department of Haematology St Vincent's Hospital Melbourne Australia; ^14^ Faculty of Medicine University of Melbourne Melbourne Australia; ^15^ Department of Haematology Liverpool Hospital Sydney Australia; ^16^ South West Sydney Clinical Campus, School of Clinical Medicine, Faculty of Medicine and Health UNSW Sydney Australia; ^17^ Department of Clinical Haematology University Hospital Geelong Geelong Australia; ^18^ Department of Haematology Gosford Hospital Gosford Australia; ^19^ Department of Haematology Gold Coast University Hospital Gold Coast Australia; ^20^ Griffith University Gold Coast Australia; ^21^ Department of Medical Oncology Grampians Health Ballarat Australia; ^22^ Department of Haematology Eastern Health Melbourne Australia; ^23^ Department of Haematology Flinders Medical Centre Adelaide Australia; ^24^ Department of Haematology Princess Alexandra Hospital Brisbane Australia; ^25^ Department of Medical Oncology and Clinical Haematology Olivia Newton‐John Cancer Research Institute at Austin Health Heidelberg Australia

**Keywords:** antibody‐drug conjugates, diffuse large B‐cell lymphoma, immunotherapy, polatuzumab vedotin, relapse, trial eligibility

## Abstract

Polatuzumab vedotin (Pola) is an approved therapy in combination with rituximab and bendamustine for relapsed or refractory diffuse large B‐cell lymphoma (RR‐DLBCL) based on positive results of the landmark phase II randomised G029365 trial. However, trial results for many approved novel therapies in RR‐DLBCL have not been replicated in routine care cohorts, as RR‐DLBCL patient populations are heterogeneous and trial eligibility is increasingly restrictive. We evaluated outcomes from pola ± bendamustine and rituximab in patients with RR‐DLBCL enrolled in a compassionate access program with no alternative treatment options identified via the Australasian Lymphoma and Related Diseases Registry according to their eligibility for the original phase II published study. Of 58 eligible patients, 74% met the criteria deeming them ineligible for the G029365 original study at the time of pola's commencement. Median progression‐free survival and overall survival in our cohort were 2.3 and 3.5 months, respectively. In contrast to the landmark trial cohort, more of our patients ceased therapy prior to completion, the majority due to progressive disease and only 8/58 received any subsequent treatment. Dismal outcomes in this Australian real‐world population demonstrate trial eligibility is challenging to meet, and newer treatments can be difficult to deliver in routine care. Clinically applicable results from therapeutic studies require trial cohorts to reflect representative clinical populations wherever possible, and more research is required to address the benefit of novel agents in the increasing majority who are ineligible for modern studies.

## 1 INTRODUCTION

Diffuse large B‐cell lymphoma (DLBCL) is the most common type of lymphoma, accounting for approximately 30%–40% of adult non‐Hodgkin lymphoma cases. Despite first‐line chemoimmunotherapy with R‐CHOP (rituximab, cyclophosphamide, doxorubicin, vincristine and prednisolone) curing the majority of DLBCL, up to 50% of patients will relapse or be refractory to initial treatment [1, 2]. Outcomes for these patients are dismal, particularly those relapsing after, or ineligible for, intensive salvage chemotherapy with autologous stem cell transplant (ASCT) [3, 4]. Even with recent survival benefits demonstrated by chimeric antigen receptor therapy (CAR‐T) delivery in fit patients with primary refractory disease [5], only 20%–30% survive long‐term. Additionally, many patients globally face challenges with CAR‐T access and suitability.

Several new agents have been approved for relapsed or refractory DLBCL (RR‐DLBCL), although most approvals are based on phase II trial results in selected populations [6, 7, 8]. With the increased restrictiveness of trials in RR‐DLBCL [9], the applicability of results to real‐world populations—typically less fit with a poorer prognosis—is often unknown. Some novel combination treatments have led to promising results within their respective registrational trials, yet significantly inferior outcomes in routine care populations who would not have met the original trial criteria. One such example is tafasitamab and lenalidomide where the median progression‐free survival (PFS) in the favourable population enrolled on the ‘L‐MIND’ trial was 12.1 months, with overall survival (OS) not reached at a median follow‐up of 17.3 months [10]. However, in the US population receiving tafasitamab‐lenalidomide in routine care, the median PFS and OS were 2.8 and 6.8 months, respectively [11]. Neither of these agents are currently approved in Australia and a treatment paradigm incorporating new agents for RR‐DLBCL has not been established.

Polatuzumab vedotin (Pola) is one such recently approved novel agent for RR‐DLBCL in several jurisdictions. Pola is an anti‐CD79b antibody‐drug conjugate with a monomethyl auristatin E payload, which binds to CD79b receptors that are near‐universally expressed on DLBCL cells [12]. CD79b is a key component of the B‐cell signalling pathway expressed on all mature B cells (aside from plasma cells) and is present in almost all B‐cell lymphomas. Pola has demonstrated activity in RR‐DLBCL as monotherapy [13] and with anti‐CD20 therapy [14] which led to evaluation in combination with bendamustine and rituximab (BR) chemotherapy [15, 16]. The landmark randomised phase II G029365 trial [15] evaluated BR with or without pola in 80 transplant‐ineligible RR‐DLBCL patients. The addition of pola to BR (Pola‐BR) demonstrated improved PFS (primary endpoint) (9.5 vs. 3.7 months; *p* ≤ 0.001) and improved OS (12.4 vs. 4.7 months; *p* = 0.002) compared to BR alone. The objective response rate (ORR) in the pola‐BR arm was 62.5% and the complete response rate was 50%. This study led to the approval of pola by the US Food and Drug Administration (FDA), and the Australian Therapeutic Goods Administration (TGA), yet is not currently reimbursed for Australian patients. Pola also now has demonstrated benefit in treatment‐naïve patients with high‐risk DLBCL [17].

Reports of Pola‐BR outcomes from RR‐DLBCL patients treated on compassionate access programs have described varied results to date [18, 19, 20, 21, 22]. Despite these including ‘real‐world’ populations, none report results according to whether patients met the eligibility criteria used by the original study. Here, we report the outcomes associated with Pola‐BR in Australian patients with RR‐DLBCL according to the presence of eligibility criteria from the G029365 trial.

## 2 METHODS

### 2.1 Study design and participants

This was a multicentre, observational study from the Australasian Lymphoma and Related Diseases Registry (LaRDR) [23]. Patients with confirmed RR‐DLBCL or high‐grade B‐cell lymphoma (HGBL) with rearrangement of *MYC* and *BCL2*/*BCL6* who received at least one cycle of pola on a compassionate access scheme in LaRDR Australian participating hospitals were included. Additional eligibility included age 18 years or above; and treated with one or more prior lines of systemic therapy.

### 2.2 Data collection

Data entered into the LaRDR by participating sites included baseline patient and disease characteristics at the time of commencement of pola as follows; stage, revised International Prognostic Index (R‐IPI) [24], Eastern Cooperative Oncology Group (ECOG) performance status, presence of bulky disease (≥7.5 cm), B symptoms, and number of extranodal sites. Treatment details collected were as follows; prior therapy, pola dosing and cycles, combination agents, response, toxicity and subsequent therapy. Categorical data were collected for most of the variables to determine eligibility criteria. Where an eligibility criterion was missing in > 20% of patients because the test was not standard care, it was not included in the analyses. Numerical data were collected for blood test results. In classifying patient eligibility, the absence of documented individual clinical characteristics (such as concomitant active infections or specific comorbidities) or abnormal laboratory results, were classified as ‘not present’.

### 2.3 Statistical analyses

The study endpoints were PFS, OS, ORR, treatment tolerability and delivery, and clinical characteristics of trial‐ineligible patients. PFS was defined as the time from pola commencement to relapse, progression or death. OS was defined as the time from pola's commencement to death from any cause. Time‐to‐event analyses were performed using the Kaplan‐Meier method, with differences between groups compared by the log‐rank test.

The response was assessed according to Lugano criteria [25]. Treatment toxicity was graded according to the Common Terminology Criteria for Adverse Events (CTCAE) version 5.0 [26].

Categorical variables were presented as frequencies and percentages with *p*‐values determined by the chi‐squared test. Continuous variables were presented as medians with 95% confidence intervals (95% CI) and p‐values determined using a rank‐sum test. Incomplete data were managed by explicitly reporting the number of records that had values for each field. Analyses were performed in Stata/BE v17.

### 2.4 Ethics approval

The ethics approval obtained from the Monash Health Human Research Ethics Committee for the LaRDR protocol (HREC/16/MonH/74) applied to this study [23].

## 3 RESULTS

### 3.1 Patient characteristics

A total of 58 patients from 19 Australian participating sites were identified from the LaRDR between 3 December 2021 and 21 February 2023. Patient characteristics at the commencement of pola‐based therapy are summarised in Table 1. The median age at the time of relapse was 63.0 years (range: 29.4–81.5 years) and 62.1% of patients were male. Forty‐five (77.6%) of the study cohort had *de novo* DLBCL and 13 (22.4%) had transformed disease. The proportion of patients who had received at least three prior lines of therapy was 54.8%.

**TABLE 1:** Patient characteristics at the commencement of pola therapy.

Characteristics	Evaluable	*n* (%)
Age at diagnosis of relapse (years), median (range)	58	63.0 (29.4–81.5)
Male gender	58	36 (62.1%)
Ann Arbor stage	49	
I–II		7 (14.3%)
III–IV		42 (85.7%)
Unknown		9
IPI score	44	
1		6 (13.6%)
2		6 (13.6%)
3		17 (38.6%)
4		11 (25.0%)
5		4 (9.1%)
Unknown		14
R‐IPI score	41	
Very good		1 (2.4%)
Good		15 (36.6%)
Poor		25 (61.0%)
Unknown		17
ECOG	49	
0–1		34 (69.4%)
2		11 (22.4%)
3		4 (8.2%)
Unknown		9
Histological subtype	58	
De novo		45 (77.6%)
Transformed from indolent lymphoma		13 (22.4%)
Presence of bulky disease (≥7.5 cm)	50	15 (30.0%)
Presence of B symptoms	51	15 (29.4%)
Number of extranodal sites	42	
1		20 (47.6%)
2		13 (31.0%)
3		2 (4.8%)
4 or more		7 (16.6%)
Unknown		16
Prior lines of treatment	53	
1		16 (30.2%)
2		8 (15.1%)
3		9 (17.0%)
4 or more		20 (37.8%)
Unknown		5

### 3.2 Pola treatment and toxicity

Data on dosing and modification of pola‐based therapy were available in 87.9% of the cohort (51/58). Of these, 30 (58.8%) received pola‐BR, 12 (23.5%) received pola with rituximab, five (9.8%) received pola with bendamustine alone and four (7.8%) received pola monotherapy. The median age of those receiving pola‐BR was 60 years, compared with 74 years for those receiving other combinations (*p* = 0.12). The details of patients requiring dose modifications are displayed in Table 2. Fifteen patients (28.3%) completed all six planned cycles of therapy, of the 53 patients with data available. Of the 38 patients who discontinued treatment prematurely, 27 (71.1%) did so due to progressive disease followed by planned bridging therapy (13.2%, *n* = 5), death (7.9%, *n* = 3) and toxicity (5.3%, *n* = 2).

**TABLE 2:** Dose modification of patients receiving pola‐based therapy.

	Pola		Rituximab		Bendamustine	
	*N*	%	*N*	%	*N*	%
Dose modification	2/51	(3.9%)	2/42	(4.8%)	5/35	(14.3%)
Full dose for the first cycle	48/48	(100.0%)	38/40	(95.0%)	30/33	(90.9%)
Full dose for six cycles	47/47	(100.0%)	38/40	(95.0%)	29/33	(87.9%)

Full dose of pola is 1.8 mg/kg once per cycle; full dose of rituximab is 375 mg/m^2^ once per cycle; full dose of bendamustine is 90 mg/m^2^ for two days per cycle.

The graded adverse events reported are described in Table 3 and presented according to the treatment received. Anaemia was reported in 70.6% and thrombocytopenia in 58.8% of the 51 patients with evaluable toxicity data. At least one planned hospital admission occurred in 50% (*n* = 25) of toxicity‐evaluable patients during Pola‐based therapy.

**TABLE 3:** Toxicity of the pola‐based treatments in all grades and grades 3–4.

	Pola (*N* = 4)		Pola‐B (*N* = 5)		Pola‐R (*N* = 12)		Pola‐BR (*N* = 30)	
Grades	All	3‐4	All	3‐4	All	3‐4	All	3‐4
Infection	0 (0.0%)	0 (0.0%)	3 (60.0%)	2 (50.0%)	3 (25.0%)	3 (25.0%)	12 (40.0%)	8 (30.8%)
Neutropenia	1 (25.0%)	0 (0.0%)	5 (100.0%)	5 (100.0%)	7 (58.3%)	4 (44.4%)	15 (50.0%)	10 (40.0%)
Anaemia	3 (75.0%)	1 (50.0%)	3 (60.0%)	3 (60.0%)	7 (70.0%)	1 (25.0%)	23 (76.7%)	11 (61.1%)
Thrombocytopenia	2 (50.0%)	1 (33.3%)	4 (80.0%)	3 (75.0%)	6 (50.0%)	4 (40.0%)	18 (60.0%)	10 (45.5%)
Peripheral neuropathy	1 (25.0%)	1 (25.0%)	0 (0.0%)	0 (0.0%)	2 (18.2%)	0 (0.0%)	9 (30.0%)	0 (0.0%)
Lymphopenia	2 (66.7%)	1 (50.0%)	5 (100.0%)	5 (100.0%)	10 (83.3%)	6 (75.0%)	23 (79.3%)	16 (72.7%)

### 3.3 Efficacy and subsequent therapy

**FIGURE 1:**
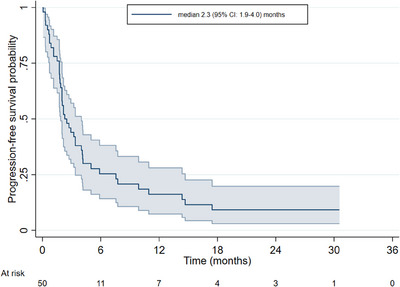
Progression‐free survival.

**FIGURE 2:**
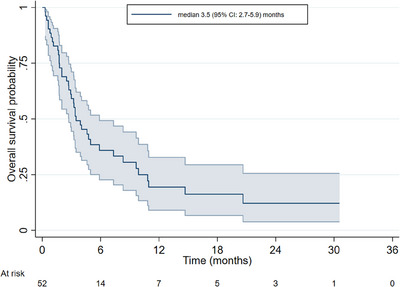
Overall survival.

The median follow‐up of the whole study cohort was 18.8 months (95% CI: 5.0–29.4 months). Median PFS was 2.3 months (95% CI: 1.9–4.0 months) (Figure 1) and median OS was 3.5 months (95% CI: 2.7–5.9 months) (Figure 2). Median OS in those receiving pola‐BR was 5.9 months (95% CI: 2.5–9.7 months) versus 3.4 months (95% CI: 1.8–4.6 months) in pola monotherapy plus pola‐R treated patients (p = 0.29). Response data were available in 48/58 patients. The ORR in evaluable patients was 45.8%, including complete response (CR) in 25% (12/48) and partial response (PR) in 20.8% (10/48). 10.4% achieved stable disease (5/48) and 43.8% (21/48) had progressive disease.

Just eight patients were reported as having subsequent lines of therapy after pola, with CAR‐T therapy delivered to two of these patients.

### 3.4 Trial eligibility

In our cohort, 43 patients (74.1%) would have been ineligible for the GO29365 study due to failing to meet at least one trial eligibility criterion, with 46.5% failing at least two eligibility criteria and 18.9% not meeting three or more eligibility criteria. Fifteen patients had no documented clinical comorbidities or abnormal laboratory results that deemed them ineligible. The most common reasons for ineligibility included the presence of a significant co‐morbidity (32.8%), being determined to be eligible by a treating physician for autologous SCT (25.9%), transformed from indolent disease (22.4%) or elevated creatinine (19.0%). Details and frequency of reasons for failure to meet eligibility are presented in Table 4.

**TABLE 4:** Distribution of GO29365 study eligibility criteria failure in our cohort.

Trial eligibility criteria	Evaluable	*n* (%)
Ineligible for the original trial^a^	58	43 (74.1%)
Number of eligibility criteria not met	58	
0		15 (25.9%)
1		16 (27.6%)
2		16 (27.6%)
3 or more		11 (18.9%)
Co‐morbidity related ineligibility		
ECOG 3–4	49	4 (8.2%)
Peripheral neuropathy > Grade 1	58	3 (5.2%)
Other specific co‐morbid conditions meeting exclusion criteria^b^	58	19 (32.8%)
Treatment‐related ineligibility		
Prior ASCT	58	9 (15.5%)
Prior AlloSCT	58	1 (1.7%)
CAR‐T cell therapy within 100 days prior to commencing pola	58	7 (12.1%)
Autologous transplant eligible	58	15 (25.9%)
Disease‐related ineligibility		
Transformed from indolent lymphoma	58	13 (22.4%)
Lesion < 1.5 cm ineligible	58	8 (13.8%)
Presence of CNS involvement	58	5 (8.6%)
Presence of active or viral infection		
Positive test results for hepatitis B viral infection	58	4 (6.9%)
Positive test results for hepatitis C viral antibody	58	2 (3.4%)
Organ or biochemical function ineligibility		
Creatinine > 135 μmol/L	58	11 (19.0%)
Haemoglobin ≤80 g/L	58	6 (10.3%)
Platelet count ≤50 × 10^9^/L	58	7 (12.1%)
Bilirubin > 30 μmol/L	58	3 (5.2%)
Alanine transaminase > 100 μ/L	58	5 (8.6%)

^a^ For eligibility criteria from the original trial where > 20% of patients had data missing due to tests not being performed, the criterion was not used for the analysis.

^b^ Significant co‐morbidities include cardiovascular, lung, kidney, and liver diseases, uncontrolled diabetes mellitus and other concomitant diseases.

Abbreviations: AlloSCT, allogeneic stem cell transplant; ASCT, autologous stem cell transplant; CAR‐T, chimeric antigen receptor T‐cell immunotherapy; CNS, central nervous system.

Of those who were categorised as eligible versus ineligible for the original study, their median PFS (1.8 months, 95%CI: 0.7–5.9 vs. 2.7 months, 95%CI: 2.0–4.1, *p* = 0.55) and OS (4.6 months, 95%CI: 1.6–10.8 vs. 3.4 months, 95%CI: 2.7–7.3, *p* = 0.97) did not reach statistical significance.

## 4 DISCUSSION

Our multicentre registry study is the first to analyse outcomes of real‐world RR‐DLBCL receiving pola ± BR according to eligibility for the original registrational GO29365 study [15] and among the largest cohorts reported to date. The median PFS and OS were dismal in our Australian population, considerably worse than the median PFS and OS of 9.2 months and 12.4 months respectively reported by Sehn et al. in the GO29365 study [15]. The substantial proportion of ineligible patients in our cohort (74%) likely contributes to this, although we found no statistically significant difference in outcomes between eligible and ineligible patients within our population. Additionally, we report an ORR which was lower than the registrational study [15], but similar to other real‐world studies – 46% (25% CR) in contrast to 63% (50% CR) in the landmark trial. The information our study provides on the treatment used with pola, number of cycles delivered, reasons for discontinuation and toxicity provides valuable insight into the practicalities of giving therapy to patients who may not fit the ideal mould of a clinical trial candidate.

Compared to the landmark trial, our patients were slightly younger (median age 63 vs. 67 years) but with a poorer ECOG (30% ECOG 2 or more vs. 15%) and nearly a third had transformed disease. Compared to the original trial [15], a much higher proportion in our cohort discontinued therapy with progressive disease (51% vs. 15%). Of note, the median OS and PFS in this Australian population were also lower than other reported real‐world studies where published survivals ranged from a median OS of 8.2–12.4 months and a median PFS between 4.0 and 9.2 months [18, 19, 20, 21, 22].

The main possible reason for the poor outcomes and tolerability in our Australian cohort is that nearly three‐quarters of our cohort were ineligible for the original GO29365 trial, with nearly half having two or more reasons for ineligibility. The more common reasons for ineligibility (such as significant co‐morbidities, elevated creatinine and hepatic dysfunction) have the effect of both prohibiting clinical trial entry as well as limiting the use of intensive salvage therapy, rendering these patients subject to few treatment options overall. Supporting this theory, approximately one‐quarter of patients enrolled in the GO29365 trial and some real‐world studies had received prior SCT compared to only 17% of our cohort [15, 19, 22]. This impacted the ability to deliver treatment too. Almost one‐third of our cohort did not receive pola in combination with both BRs, despite the Therapeutic Goods Australia licensing approval being based on the combination therapy with these agents. Furthermore, 71.7% did not receive all six planned cycles despite an absence of progressive disease in many. Concerns from clinicians regarding the tolerability of bendamustine and challenges in the ability to deliver some treatments to real‐world RR‐DLBCL cohorts are clearly reflected in these results.

The number of cycles delivered and the lower rate of combination treatment similarly appeared to affect outcomes in two other studies. The first was a study of 40 patients by Wang et al. [22] where a median of only three cycles was delivered, and just 53% of patients received pola with BR. The reported ORR of 53% (CR in 25%) is very similar to our study. However, median OS was higher in this group, (8.5 months), with an improved OS (24.0 vs. 4.4 months) in those who were successfully bridged to haematopoietic SCT. The second by Smith et al. [27] also reported an ORR of 50% and CR of 24%, with a median of just two cycles of pola‐based therapy. In this study, median PFS and OS were likewise poor (2.0 and 5.3 months), despite a higher proportion of patients who were bridged to other therapies, the majority being CAR‐T therapy. These are not dissimilar to results from the original monotherapy [13] and pola‐rituximab [14] trials where the reported median PFS was 5.6 months and ORR of 54% (21% CR rate) in heavily pre‐treated and commonly refractory DLBCL patients.

This study has several limitations associated with its retrospective, registry‐based nature, and the sample size available. The population was heterogeneous and the timing of response assessments was not standardised. Detailed information on reasons for the use of alternatives to full dose pola‐BR, subsequent dose reductions and cessation, therapy intent and subsequent therapies was not complete and the study was not powered to detect differences in outcome according to individual failed eligibility criteria. The large number of patients with at least one reason for ineligibility meant that we were unable to detect a difference in outcomes between those meeting eligibility, versus those who did not. A larger cohort with increased statistical power is required to draw any direct inferences regarding associations of outcomes with being eligible.

The poor outcomes of our cohort may have been due to 74% having features which would have deemed them ineligible for the original trial. It is also noteworthy that these patients participated in a compassionate access program, and thus may have been ineligible for other clinical trials and more intensive therapies due to their poorer risk profile. This is supported by the higher proportion of heavily pre‐treated patients with three or more prior lines of therapies in our cohort of patients enrolled in the compassionate access programs compared to the patients in the original trial (54.8% vs. 45%).

Given the newer available therapies globally, it is critical to ensure these agents benefit the population represented in clinical care. Thus, further data from relevant populations on which poor‐risk disease and patient features are associated with poor outcomes with these agents is needed. These data can inform how pola and other agents fit into the landscape of therapeutic options, and how to improve guidance on supportive care (e.g. prophylactic growth colony‐stimulating factors) and monitoring to further bolster the number of patients able to tolerate and benefit from six complete cycles of pola‐BR.

## 5 CONCLUSION

Our analysis demonstrates dismal outcomes from pola‐based treatment in RR‐DLBCL compared to the registrational GO29365 study in a population where the majority of patients failed to meet the landmark study eligibility criteria. This real‐world Australian population experienced early discontinuation and was frequently unable to receive subsequent therapy. Discordant results between registrational clinical trials and routine care are increasingly common, risking the creation of unrealistic expectations and treatment in the context of futility. The importance of recruiting representative populations to clinical trials to ensure results are relevant to the majority of routine care patients, and communication of accurate expectations to patients is imperative in future research. Further studies in diverse cohorts and in other combinations are essential.

## AUTHOR CONTRIBUTIONS

Eliza A. Hawkes, Eliza Chung, Cameron Wellard, Kate Manos and Briony Shaw designed the study, secured funding and oversaw the data collection, and analysis and wrote the manuscript. Edward Yoo, Rory Bennett, Callum Birks, Anna Johnston, Chan Y Cheah, Nada Hamad, Jock Simpson, Allison Barraclough, Matthew Ku, Nicholas Viiala, Sumita Ratnasingam, Tasman Armytage, Tara Cochrane, Geoffrey Chong, Denise Lee, Colm Keane, Stephanie Wallwork and Stephen Opat contributed to data collection and analyses. All authors approved the final manuscript.

## CONFLICT OF INTEREST STATEMENT

Briony Shaw, Eliza Chung, Edward Yoo, Rory Bennett, Callum Birks, Jock Simpson, Sumita Ratnasingam, Tasman Armytage, Denise Lee, Colm Keane, Stephanie Wallwork all declare no conflicts of interest. Anna Johnston: Consulting or advisory role, honoraria: Merck Sharpe & Dohme, Roche, Link, BeiGene, Sanofi, EUSA Pharma, Novartis, Chan Y Cheah: Consulting or advisory role, honoraria: Roche, Janssen, Gilead, AstraZenecca, Lilly, TG therapeutics, BeiGene, Novartis, Menarini, Dizal, AbbVie, Genmab, Bristol Myers Quibb; Research funding: Bristol Myers Quibb, Roche, AbbVie, Merck Sharpe & Dohme, Lilly, Nada Hamad: Honoraria: Roche, Janssen, Gilead, AstraZenecca, BeiGene, Novartis, AbbVie, Genmab, Bristol Myers Quibb, Takeda, Jazz Pharmaceuticals, Incyte, Pfizer, Mallinckrodt Pharmaceuticals, Terumo, Allison Barraclough: Honoraria: Roche, Gilead, Novartis, BeiGene, Matthew Ku: Roche, Antengene, Genor BioPharma, Nicholas Viiala: Honoraria and advisory board: Novartis, Tara Cochrane: Consulting or advisory role: Janssen; Honoraria: Celgene (in 2019); Research funding: BeiGene, Geoffrey Chong: Research funding: Bristol Myers Quibb, Merck, AstraZeneca, Pharmacyclics, Regeneron, Hutchmed, Bayer, Incyte, Amgen, Kate Manos: Advisory/honoraria: AbbVie; Research funding: Roche, Stephen Opat: Honoraria: AbbVie, BeiGene, AstraZeneca, Bristol Myers Quibb, CSL Behring, Gilead, Janssen, Merck, Roche, Takeda; Research funding: AbbVie, BeiGene, AstraZeneca, CSL Behring, Gilead, Janssen, Merck, PharmaCytics, Roche, Takeda, Eliza A. Hawkes: Consulting or advisory role: Roche, Merck Sharpe & Dohme, AstraZeneca, Gilead, Antengene, Novartis, Regeneron, Janssen, Specialised Therapeutics; Research funding: Roche, Bristol Myers Squibb, Merck KGaA, AstraZeneca, Merck

## ETHICS STATEMENT

This study is conducted under the ethics approval obtained from the Monash Health Human Research Ethics Committee for the LaRDR protocol (HREC/16/MonH/74).

## PATIENT CONSENT STATEMENT

All patients recruited in this study had provided consent.

## CLINICAL TRIAL REGISTRATION

The authors have confirmed clinical trial registration is not needed for this submission.

## Data Availability

The data that support the findings of this study are available on request from the corresponding author. The data are not publicly available due to privacy or ethical restrictions.
